# Supplementary material for: Genome-Wide Analysis of Flax (Linum usitatissimum L.) Growth-Regulating Factor (GRF) Transcription Factors
Source: Int J Mol Sci. 2023 Dec 4;24(23):17107. doi: 10.3390/ijms242317107 (PMC10707037; doi:10.3390/ijms242317107)
Supplement: Supplementary file 1 [file ijms-24-17107-s001.zip › Supplementary Figure S1.pdf]

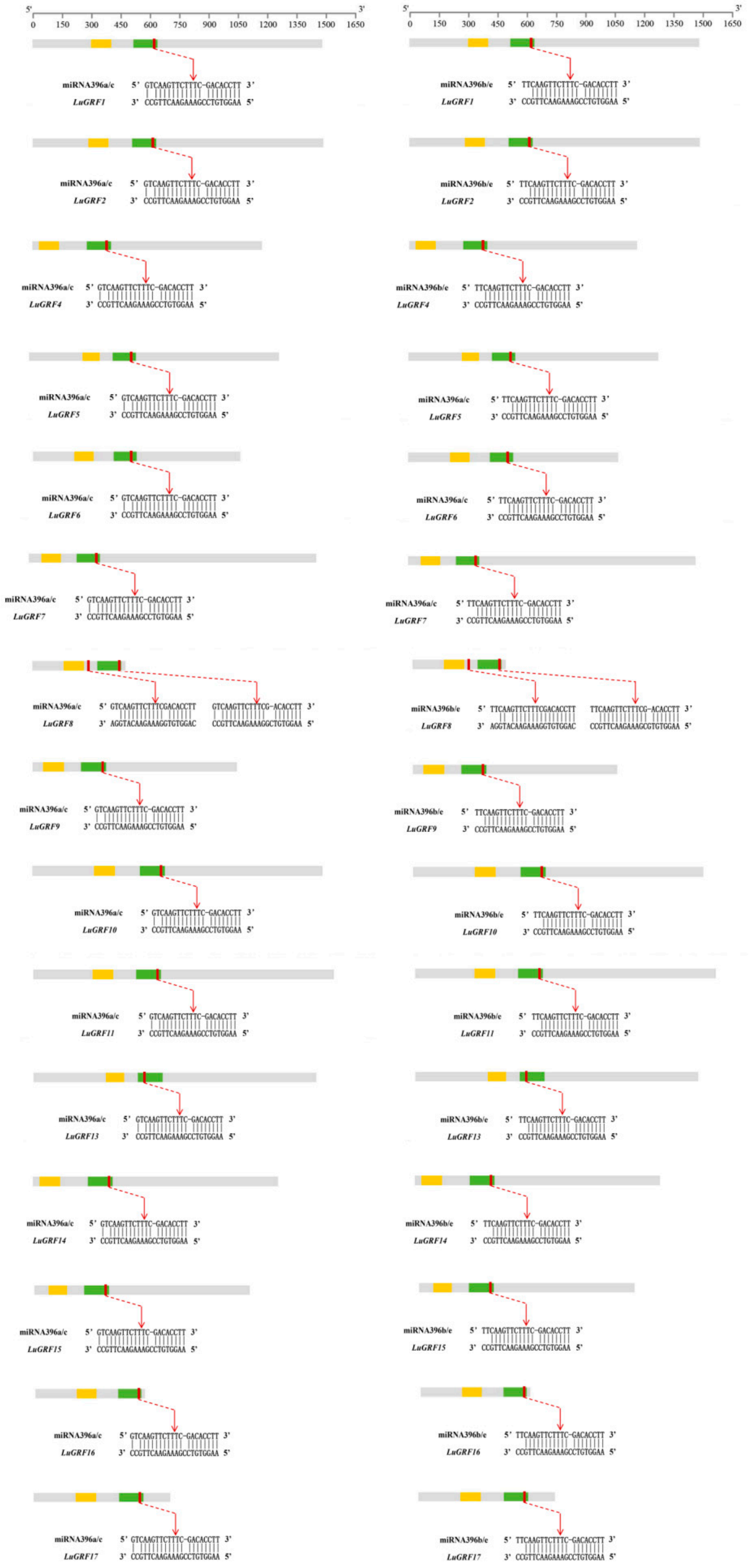

Figure S1. The target location of miRNA396 in the *Lu*GRF gene family. The yellow box represents the QLQ conserved domain, the green box represents the WRC conserved domain, and the gray box represents the CDS region of the GRF gene.
