# Supplementary material for: Genome-Wide Analysis of Flax (Linum usitatissimum L.) Growth-Regulating Factor (GRF) Transcription Factors
Source: Int J Mol Sci. 2023 Dec 4;24(23):17107. doi: 10.3390/ijms242317107 (PMC10707037; doi:10.3390/ijms242317107)
Supplement: Supplementary file 1 [file ijms-24-17107-s001.zip › Supplementary TableS1.pdf]

Table S1. The primers used in this experiment

Gene name	Forward primer	Reserve primer
qLuGRF1	GTCAGTTGGGCGTTACAGT	GACCGCTACTCCTGCTCCT
qLuGRF2	GGAGCAGTTTGAATGTGAC	CTTCTTGATGGGATTGAGC
qLuGRF3	TCCAAAGGGTACTACTGCT	AAAGGCTTCTGGGTTTCAT
qLuGRF4	TTACAGGAGCATTTACGGG	AGTTTATTGCCAGCGGACT
qLuGRF5	TCATAAGCCTAATCTCCAA	CTTCACGACCCATTCCAAA
qLuGRF6	CAGCAACAGCAGCACAATA	AATCAGCAAACAAAGGGAG
qLuGRF7	CTATGGGAAGAAGGTGGAT	ACAATACTTGGAGTCAGGGT
qLuGRF8	ACCAGGGAGGTGTAGACGA	TTTCTTGAACGGTTGAGGC
qLuGRF9	GAAAGCCAGTCGCTTCGTC	ACCTGCTGCTGTCATCGTG
qLuGRF10	CCTTCATCCCGAGTCATTT	CATTTCTTCCCATCTGTCC
qLuGRF11	ATTAGCTGGCCCGAAGAGC	CTGGCGATGAGGACGAGGA
qLuGRF12	GCCTCCCAATCCCACCTCA	CTGCACCTCCCTGGCTCAA
qLuGRF13	CATGGGCAGACATTCGTTG	GACATCCCTGGAGCACCTC
qLuGRF14	CCTCCTTCTGGATCTTACA	GTCATTAGTCTGTTGTGGC
qLuGRF15	TTGTATTGGGTGCTGATTT	ATGGTGGTGATATTGATTGAC
qLuGRF16	AACCATTGGACTGGGACTA	CTCTTGAATCTGCGACACT
qLuGRF17	AGAACAGCAACAGCAGCAC	TGAATCAGCAAACAGAGGG
pLuGRF1	TCTCATTGCCATTCCGAGTTTG	ACATCGTCGCAGAGGCTATTCC
pGD-LuGRF1	TCTCTCTACAAGATCTCGAG	CCC TTG CTC ACC ATG TCG AC
	TCTCATTGCCATTCCGAGTTTG	ACATCGTCGCAGAGGCTATTCC
GAPDH	CTTTACCCTCAGCAAATCCG	AGGTTCTTCCCGCTCTCAAT
