# Supplementary figures and images for: Genome-Wide Analysis of Flax (Linum usitatissimum L.) Growth-Regulating Factor (GRF) Transcription Factors
Source: Int J Mol Sci. 2023 Dec 4;24(23):17107. doi: 10.3390/ijms242317107 (PMC10707037; doi:10.3390/ijms242317107)

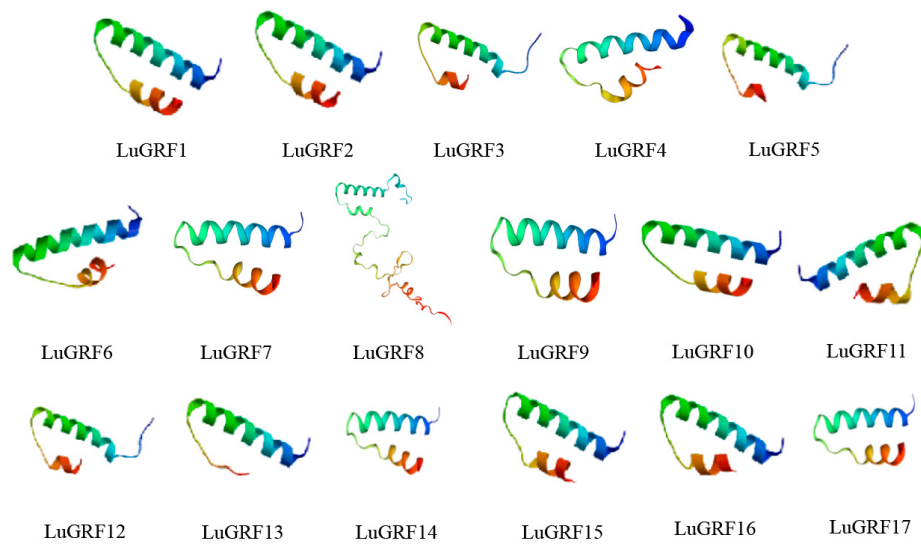

Figure S2. 3D structural modeling of protein encoded by LuGRF gene.

Supplement: Supplementary file 1 [file ijms-24-17107-s001.zip › Supplementary Figure S2.pdf]
